# Rice Bran Stabilisation and Oil Extraction Using the Microwave-Assisted Method and Its Effects on GABA and Gamma-Oryzanol Compounds

**DOI:** 10.3390/foods11070912

**Published:** 2022-03-22

**Authors:** Núria Reis, Ana Castanho, Manuela Lageiro, Cristiana Pereira, Carla Moita Brites, Manuela Vaz-Velho

**Affiliations:** 1 CISAS—Center for Research and Development in Agrifood Systems and Sustainability, Instituto Politécnico de Viana do Castelo, Rua da Escola Industrial e Comercial Nun’Alvares 34, 4900-347 Viana do Castelo, Portugal; mvazvelho@estg.ipvc.pt; 2 Edius-International PhD School of the USC, Department of Analytical Chemistry and Food Science, Faculty of Science, University of Santiago de Compostela, 27002 Lugo, Spain; 3 GreenUPorto—Sustainable Agrifood Production Research Centre & DGAOT, Faculty of Sciences, University of Porto, Campus de Vairão, Rua da Agrária 747, 4485-646 Vairão, Portugal; anavargascastanho@gmail.com; 4 National Institute for Agricultural and Veterinary Research (INIAV), I.P., Av. da República, 2780-157 Oeiras, Portugal; manuela.lageiro@iniav.pt (M.L.); cristianapereirags@gmail.com (C.P.); carla.brites@iniav.pt (C.M.B.); 5 GeoBioTec, Nova School of Science and Technology, 2829-516 Caparica, Portugal; 6 GREEN-IT Bioresources for Sustainability, ITQB NOVA, Av. da República, 2780-157 Oeiras, Portugal

**Keywords:** rice bran oil, microwave, gamma-aminobutyric acid, gamma-oryzanol, fatty acids

## Abstract

Rice bran oil (RBO) is a valuable ingredient extracted from rice bran (RB), a side stream of polishing rice grain in the milling process. RBO is rich in bioactive ingredients with potential health benefits, such as gamma-oryzanol (GO) and gamma-aminobutyric acid (GABA). Despite its benefits, the quality of RBO depends on the degree of stabilisation of the RB, which is easily affected by lipase enzymes, and thus needs an effective treatment prior to RBO production. To assess the potential of the microwave-assisted method for RB stabilisation and RBO extraction, three Carolino rice varieties (Ariete, Teti, Luna) were tested. The effect of RB stabilisation was evaluated via acid value, water absorption, and GO and GABA levels. The RBO yield was optimised by solvent, temperature, and solvent-to-sample ratio, and the GO and fatty acid levels were determined. The RB stabilisation for the Luna variety did not affect the GO and GABA; for the Ariete and Teti varieties, the GO decreased by 34.4% and 24.2%, and the GABA increased by 26.5% and 47.0%, respectively. The GO levels in RBO samples were not affected by RB stabilisation. The RBO nutritional value was confirmed by the suitable ratio (>2) between polyunsaturated (PUFA) and saturated fatty acids (SFA), with the Teti variety presenting the highest ratio.

## 1. Introduction

Rice (*Oryza sativa L.*) is one of the most globally consumed cereals and is a staple food. Usually, rice is consumed after dehulling and milling and consists of 70% of the whole paddy grain; the other 30% are side streams of the process and includes husk, bran, and germ. Rice bran (RB) accounts for 8% of the grain [1] and, despite being rich in protein (17–30%) and oil (20–23%), and a source of bioactive compounds [2], rice bran is still used mainly as animal feed [3]. Rice bran oil (RBO) has gained popularity in recent years due to its optimal combination of polyunsaturated and monounsaturated fatty acids, and the presence of tocopherols and gamma-oryzanol (GO) compounds [4].

GO is a mixture of ferulate esters of sterols and triterpene alcohol, including 24-methylenecycloartanyl ferulate (MCAF), cycloartenyl ferulate (CAF), campesteryl ferulate (CampF), and β-sitosteryl ferulate (SF) [5,6,7]. GO is considered a promising compound and has been studied for its health benefits, mainly regarding lipid metabolism regulation [8,9,10], cardioprotective effects [11], and glucose metabolism regulation [12]. In addition, the potential role of GO against cancer has also been studied in cell lines [13,14] and animal models [15]. GO is found in RB at a concentration ranging from 59.4 to 912.0 mg/100 g [14] and from 100.0 to 2410.0 mg/100g in RBO [16], depending on factors such as the extraction method and rice bran origin.

RB also contains gamma-aminobutyric acid (GABA). The role of GABA in human health, the hypothesis of GABA supplementation, and its potential benefits have been investigated [17]. This non-protein amino acid acts as the primary neurotransmitter in the human cortex. It is responsible for the balance between the excitation and inhibition of neurons by binding to specific membrane proteins [18]. The presence of GABA is ubiquitous in the human diet, as it can be found in plants, animals, and microorganisms [19]. GABA is synthesised through the glutamate decarboxylase (GAD) enzyme, which catalyses the α-decarboxylation of L-glutamic acid to produce GABA and carbon dioxide [20]. GAD and GABA are present in rice, mainly in the germ fraction, and their content in bran is also relatively high [21]. The amount of GABA in rice bran ranges from 10.7 to 350.0 mg/100 g. Increased levels of GABA can be obtained by reinforcing the germination status of the grain [22]. GAD activity and, consequently, GABA content can be affected by stressing conditions during rice growth, such as anaerobiosis, pH, and temperature [21]. 

Despite the value of its compounds, RB production for human consumption can be hampered by its rapid oxidation, as it is easily affected by lipase enzymes, leading to the hydrolysis of triglycerides into glycerol and free fatty acids (FFA). An excess of FFA (5%) makes the oil less profitable and even not suitable for human consumption (10% FFA); it is, therefore, essential to inactivate the lipase enzymes before any use of the RB [23]. The lipase enzymes in RB can be inactivated by chemical [24] or thermal treatment. There are several methods reported in the literature for RB stabilisation by heating, including parboilisation [25], sub-critical water treatment [26,27], dry heating [28,29], extrusion [30], ohmic heating [31], infrared heating [32], and microwave heating [33]. 

Along with its role in RB stabilisation, microwave treatment also improves the subsequent oil extraction. Tao et al. [23] reported an improvement in oil yield after the microwave stabilisation procedure, observing an agglomeration of RB after the treatment. Other authors [28,34] have also reported an increased oil yield in stabilised rice bran (SRB). This result can be explained by the cell wall disruption caused by the high inner pressure due to the rapid heating of water molecules [35].

Due to the reduced extraction time, energy, and solvent consumption, microwave-assisted extraction (MAE) of fats and oils has gained popularity [36]. One of the factors contributing to the success of MAE is rapid heating, as it contributes to a quick extraction and increases the oil yield. Furthermore, it is an accurate method as the temperature can be controlled, and the solvent can be quickly heated over its boiling point, requiring less energy and solvent [37]. However, despite the clear advantages of using microwaves for RB stabilisation and oil extraction, this process may impact the quantity of bioactive compounds. Therefore, this study aims to quantify GO and GABA contents in RB from three Carolino rice varieties (Ariete, Teti, Luna) and understand the impact of microwave-assisted stabilisation and oil extraction on these bioactive compounds. 

## 2. Materials and Methods

### 2.1. Chemicals

HPLC grade methanol, acetonitrile, and isopropanol were obtained from Chem-Lab (Zedelgem, Belgium), while phenol and GABA standards were obtained from Sigma Aldrich (St. Louis, MO, USA). Analytical grade anhydrous ethanol and sodium hydroxide were purchased from Thermo Fisher Scientific (Waltham, MA, USA). GO standards were obtained from TCI Europe (Zwijndrecht, Belgium).

### 2.2. Rice Bran (RB) Samples

Three commercial Carolino rice cultivars (*Oryza sativa* L. ssp. *japonica*, long grain with intermediate amylose contents) were tested, Ariete, Luna, and Teti, grown in Portugal in the Mondego, Sado, and Tejo river valleys, respectively. Rice bran was supplied by an industrial mill and was obtained by vertical polishing abrasion of the brown rice, with the bran removed afterwards by aspiration. The samples were stored at −18 °C in a vacuum-sealed plastic bag PA/PE 20/70 with an O_2_ permeability of 50 cm^3^/m^2^ dbar (Termofilm, Portugal) until analysis. On the day of the stabilisation procedure, the samples were thawed for four hours at room temperature and then sieved to a maximum size of 710 µm. The rice bran used for stabilisation tests and oil extraction was obtained from different batches.

### 2.3. Rice Bran (RB) Stabilisation

RB stabilisation was performed according to the procedure described in Malekian et al. [38]; the RB was hydrated to 21%. For three minutes, batches of 150 g were placed in sealed polyethylene bags and exposed to conventional microwave heating (2.450 MHz and 550 W). The SRB was cooled (from 107 °C) to room temperature. The samples were stored in the bags used for stabilisation, closed, at room temperature, and protected from daylight until analysis.

### 2.4. Stability Evaluation Tests

Stability evaluation tests (acid value, water absorption capacity, and moisture content) were performed eight times over 16 weeks (T0 to T7) in stabilised rice bran (SRB) and non-stabilised rice bran (NSRB).

#### 2.4.1. Acid Value (AV) Analysis 

The AV analysis was performed according to ISO 7305:2019 [39]. Five grams of RB was diluted in 30 mL of anhydrous ethanol, stirred for 60 min, and centrifuged at 5000 rpm. The supernatant (20 mL) was titrated with sodium hydroxide (NaOH), using phenolphthalein as an indicator. The results are expressed as mg NaOH per 100 g of dry matter.

#### 2.4.2. Water Absorption Capacity (WAC)

The WAC analysis was performed according to Sosulski [40] with minor modifications. Five grams of RB was suspended in 30 mL of water; the mixture was stirred 7 times with a 10 min interval, centrifuged at 2300 rpm for 25 min, and the residue was dried. WAC was calculated as grams of water absorbed per 100 g of dried pellet.

#### 2.4.3. Moisture Content (MC)

The MC determination was performed according to ISO 6496:1999 [41]. Five grams of RB was weighed into ceramic crucibles and dried in an oven (103 ± 2 °C) until a constant weight was reached (about 150 min).

### 2.5. Optimisation of Microwave-Assisted Extraction (MAE)

The MAE method was based on the Zigoneanu et al. [42] procedure and optimised in order to maximise the oil yield. 

The MAE was performed at three different temperatures (80 °C, 100 °C, and 120 °C) for 15 min; two types of solvents (ethanol, isopropanol) and two solvent-sample ratios (3:1; 9:1). The temperatures and solvent ratios were chosen based on the bibliography and preliminary tests using a range of temperatures (40 °C to 120 °C) with a solvent ratio of 3:1.

Before running the extraction program of the MAE, samples of 5 g of SRB and NSRB were weighed into the Teflon vessels of the Ethos X Microwave Extraction System (Milestone Inc., Monroe, CT, USA). Samples were cooled and filtered to remove solid particles. Crude RBO was obtained by removing the solvent using a rotary evaporator (BUCHI, R-124 rotary evaporator with B-480 waterbath, Flawil, Switzerland).

### 2.6. Gamma-Oryzanol (GO) Quantification

The GO quantification was performed by reverse-phase high-performance liquid chromatography (HPLC) with a diode array detector (DAD), according to the methodology reported by Lageiro et al. [43] with minor modifications. The RB lipid residue was extracted according the procedures reported by Castanho et al. [14], and the extract was dissolved in 5 mL of methanol; the RBO was dissolved in 3 mL isopropanol and filtered through a 0.22 μm nylon syringe filter (Pall Corporation Europe, Portsmouth, Hampshire, UK) before the injection. The chromatographic system was an HPLC Agilent Technologies 1200 series (192 HPL, Agilent Technologies, Santa Clara, CA, USA), with a quaternary pump (G1311A), autosampler (ALS G1329A), column oven (TCC G1316A), DAD (G1315B), a reverse phase C18 column (Spherisorb ODS2 150 mm × 4.6 mm, 5 µm particle size, Waters Corporation, Milford, MA, USA), and a C18 precolumn (AQC18 4 mm × 3 mm, Phenomenex, Torrance, CA, USA) operating in the isocratic mode with a mobile phase of acetonitrile:methanol (50:50 *v*/*v*) during 25 min with a flow rate of 1.2 mL/min and injection volumes of 20 µL. Solvents were filtered with a 0.22 µm nylon membrane and ultrasound-degassed for at least 20 min. The GO compounds identification was made at 325 nm by retention times and spectra comparisons with GO standards from TCI Europe. The GO quantification was based on an external calibration curve with a GO standard (10 to 900 mg/L). A linear regression between the total GO area (peak area sum of the different GO compounds peak areas) and the GO content, [GO], in µg/mL, was obtained (total GO area = 29.10 × [GO] − 58.99) with a determination coefficient of 0.9995. The RB limit of quantification (RB-LQ) was 23.9 mg/100g of RB sample (using 0.8 g RB sample and 5 mL extraction volume), and RBO-LQ was 229.0 mg/100g of RBO sample (using 0.05 g RBO sample and 3 mL extraction volume). The GO compounds MCAF, CAF, CampF, and SF percentages were obtained by the ratio of the peak area of each compound to the total GO area.

### 2.7. Gamma-Aminobutyric Acid (GABA) Quantification

The GABA contents in RB were determined using the colorimetric method described previously by Zhang et al. [44], using 6% phenol and 9% alkaline hypochlorite reactants. GABA was extracted with 5 mL of deionised water centrifuged at 4500 rpm (10 min, 4 °C); the supernatant was filtered and collected for analysis. The obtained samples were measured at a wavelength of 645 nm in a UV-visible spectrophotometer (U-2010, HITACHI Ltd., Tokyo, Japan). GABA quantification was based on an external calibration curve with a GABA standard (0.01 to 0.2 mg/mL). 

### 2.8. Fatty Acid Composition

The fatty acid composition of the stabilised rice bran oil was determined with gas chromatography according to ISO 12966-2:2017 [45]. An Agilent 7820A gas chromatographer was used with a Supelco SP-2380 60 m × 0.25 mm × 0.2 µm column.

**Figure 1:**
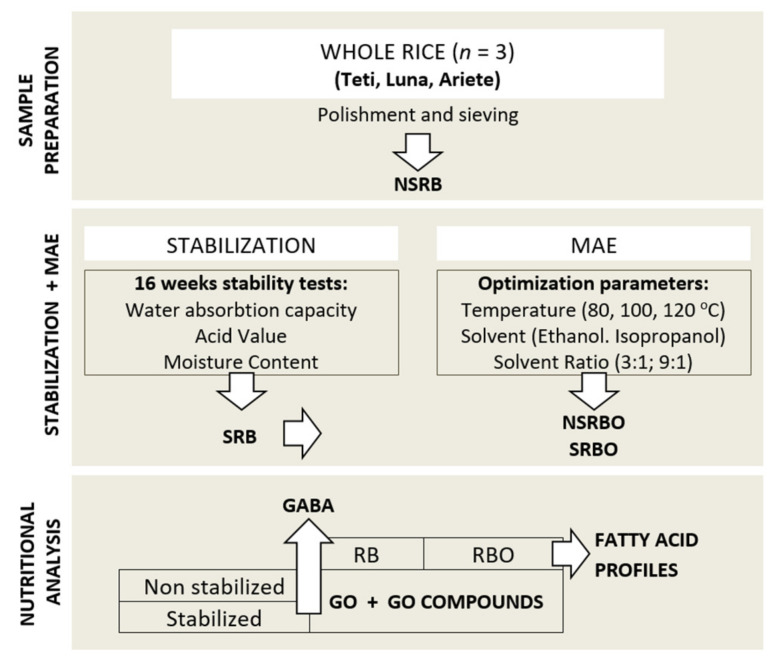
Flow diagram showing the sequence of experimentation, analyses, and methodologies. NSRB: non-stabilised rice bran; SRB: stabilised rice bran; MAE: microwave-assisted extraction; NSRBO: non-stabilised rice bran oil; SRBO: stabilised rice bran oil; GO: gamma-oryzanol; GABA: gamma-aminobutyric acid.

The general scheme of the sequence of experimentation, analyses, and methodologies used is presented in Figure 1.

### 2.9. Statistical Analysis

All analyses were performed in triplicate. Statistical data were analysed through SPSS v26 (SPSS Inc., Chicago, IL, USA) by Duncan’s multiple range ANOVA and the Mann–Whitney U-test, considering *p* < 0.05 as statistically significant.

## 3. Results and Discussion

### 3.1. Rice Bran Stabilisation Parameters

The water absorption capacity (WAC), moisture content (MC), and acid values (AV) of the SRB and NSRB for the initial and final storage times are shown in Table 1. The RB stabilisation was monitored mainly by AV data. Despite the fact that WAC and MC are not stabilisation indicators, they are important quality control parameters for the food industry and were monitored for 16 weeks.

Regarding the WAC, the values of the NSRB decreased significantly (*p* < 0.05) over time in the Ariete and Teti varieties; on the other hand, there was a noticeable and significant WAC increase (*p* < 0.05) in SRB samples. There were no changes in the WAC in the Luna variety over time for NSRB or SRB. WAC values depend on many factors such as the size, shape, and hydrophilic-hydrophobic balance of amino acids in protein molecules, and their thermodynamic properties [38]. Malekian et al. [38] reported that microwave-heat-stabilised and extrusion-stabilized samples showed higher WACs than controls, with a significant decrease (*p* < 0.05) during storage. A different tendency was observed in this experiment; the WAC of two stabilised rice bran varieties increased during storage time, probably due to the absence of extrusion. 

The MC decreased over time in all the NSRB samples (*p* < 0.05). The same tendency was verified in the SRB of the Luna variety. On the other hand, SRB from the Ariete variety shows a significant increase (*p* < 0.05) in MC. No differences were found in the MC for Teti SRB.

The effect of stabilisation on the RB can also be noticed: the treatment led to statistically significant differences (*p* < 0.05) in all values of WAC and MC when comparing the initial and final times for each variety. Ramezanzadeh et al. [46] also reported higher moisture content in the SRB initially than in the NSRB samples. The addition of water to the samples increases the ionic conductivity, contributing to faster electromagnetic energy absorption and a higher heating rate [47]. Although the moisture increment might lead to an inefficient inactivation of lipoxygenase, Ramezanzadeh et al. [46] found that its activity decreased due to the lack of FFA, the substrate for lipoxygenase activity.

**Figure 2:**
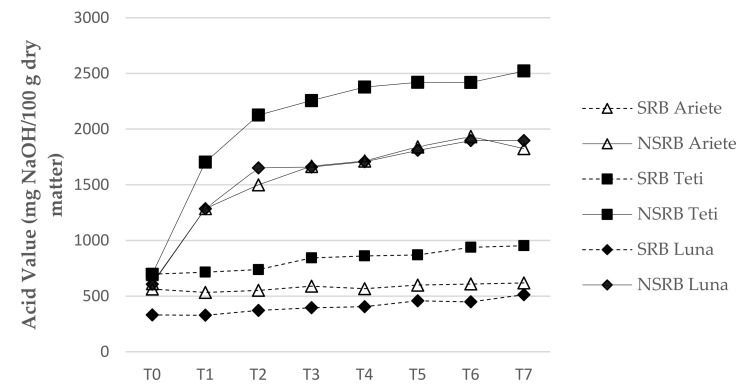
Acid value evolution along storage time from initial measurement T0 to final measurement T7 (week 16), expressed in mg NaOH/100 g dry matter.

The AVs of the RB samples during storage are presented in Figure 2. There were highly significant differences between NSRB and SRB samples along the storage period; in fact, the values determined for T0 already demonstrated significant (*p* < 0.05) differences for the Teti and Luna varieties. These differences are even higher after this period: the AVs of the NSRB samples from T1 to T2 increased markedly, reaching 2500 mg NaOH/100g dry matter on week 16 (T7). There was a slight increase in the AVs of SRB samples during storage but these never exceeded (1000 mg NaOH/100 g dry matter). Lavanya et al. [33] have also reported the increase in the AV of stabilised RB at a lower rate during 30 days of storage. Thanonkaew et al. [28] tested domestic methods for RB stabilisation (MW, hot air, roasting, and steaming) and reported a better performance of microwave stabilisation based on the decrease in the AV, and both free fatty acids and peroxide values were significantly lower after stabilisation. High levels of unsaturated free fatty acids and the presence of lipoxygenase favour lipid oxidation. Additionally, high moisture levels may further enhance the enzymic oxidation, and exposure to heat, oxygen, catalysts, or light accelerates nonenzymic oxidation [48]. Galliard [49] also found that during storage, there is a relatively slow (over several weeks) release of fatty acids, catalysed by a lipolytic enzyme in the bran. Still, lipid oxidation occurs rapidly (within minutes) when excess water is added, facilitating lipoxygenase-catalysed peroxidation of polyunsaturated fatty acids.

### 3.2. Rice Bran Oil (RBO) Extraction Optimisation by the MAE

The results of RBO extraction by the MAE are shown in Table 2. Three temperature conditions (80 °C, 100 °C, and 120 °C), two solvent-to-sample ratios, and two solvents were tested for each RB variety. The highest extraction yields (18.20%, 17.90%, 17.88%, and 17.27%) were obtained at 120 °C, using ethanol with a 9:1 solvent-to-sample ratio. These values are in line with those registered by Kanitkar et al. [35], who experienced a maximum oil yield of 17.2% with ethanol at 120 °C when using the MAE, compared to 12.4% using a reflux system under the same conditions for the extraction of RBO. The extraction yield of ethanol was 13.6%, 3.5%, and 11%, respectively, for the Teti, Luna, and Ariete varieties. 

A four-way ANOVA was performed to compare the main effects of variety, solvent, solvent-to-sample ratio, and temperature on oil yield (results not shown). All factors except variety were statistically significant at *p* < 0.05. Temperature showed the largest effect size, explaining 64% of the variance, followed by the solvent-to-sample ratio (23%) and the interaction of these two factors (9.7%).

The results, presented in Table 2, show that the oil yield is higher when the sample is heated to 120 °C. Additionally, the 9:1 ratio has a better extraction performance, and ethanol proved to be the most suitable solvent for the MAE compared to isopropanol, as the highest yields were obtained with ethanol. Zigoneanu et al. [42] tested different temperatures (40 °C to 120 °C) and solvents (isopropanol and hexane) via the MAE. The authors found that at 120 °C, the oil yield was 50% higher when using isopropanol as an extraction solvent as compared to hexane. This can be attributed to the higher solubility of RBO in isopropanol, which could lead to the extraction of more polar components such as alcohol-soluble proteins and carbohydrates, therefore increasing the crude amount of oil extracted.

The RBO used for the subsequent analyses (GO and fatty acid profiles) was extracted using ethanol at a 9:1 ratio and a temperature of 120 °C, as these conditions conferred higher extraction yields. In those conditions, the yield did not show statistically significant differences between varieties at *p* < 0.05.

### 3.3. Rice Bran Oil Fatty Acid Composition

The fatty acid profiles of stabilised RBO are shown in Table 3. The main fatty acids in RBO are oleic (C18:1n9c), palmitic (C16:0), and linoleic (C18:2n6c) acids. Other authors [50,51,52,53,54] have also reported the same acids as the major constituents in RBO. The essential fatty acid C18:3n3c (linolenic) was present in all RBO samples, with Teti samples showing higher contents. Actually, the percentage of linolenic acid in all Carolino RBO samples was higher than that reported for other RBO [50,51,52,53,54] and other vegetable oils, being even higher than in olive oil [55]. There were statistically significant differences in some fatty acids contents, the most evident occurring in palmitic and linoleic acids, with the Luna and Teti varieties presenting higher contents than Ariete. The analysed samples showed a PUFA:SFA ratio > 2, higher than those reported by other MAE–RBO studies [50,51,56], with Teti RBO presenting the highest ratio. Previously reported randomised controlled trials have evidenced that the substitution of SFA intakes with PUFA decreases cardiovascular and coronary heart disease events [57]. Thus, RB oil from the studied Carolino varieties can be considered healthier, with the Teti RBO variety showing the greatest health-claim potential among the tested varieties. 

### 3.4. GO and GABA Contents

The results shown in Table 4 reflect that Teti had higher levels of GO and GABA, but also that the microwave stabilisation process can affect the GO and GABA contents in RB. There was a statistically significant decrease (*p* < 0.05) in the GO content after stabilisation in the Ariete and Teti varieties (about 34.4% and 24.2%, respectively); the Luna variety did not show any significant differences. The different GO contents among cultivars and the different results when extracted may be related to the rice origin or genetic factors. Other authors [43] have explored the differences between the GO values of RB samples from different cultivars grown in different environments, reporting that genetic and environmental factors may contribute to the differences in GO contents. 

The varieties also presented different results regarding the level of GABA content after stabilisation. There was a significant increase (*p* < 0.05) in the GABA content in the Ariete and Teti varieties after the stabilisation process (about 26.5% and 47.0%, respectively), while the Luna variety did not show significant differences. These results reveal that the GABA values after stabilisation may also depend on genetic factors. 

The changes in the GO and GABA contents may also reflect the impact of the stabilisation process, mainly the temperature. High temperatures or heating food processes can affect food in different ways; the modification of cell structure may occur, enabling molecular interactions or even availability, formation, or deterioration of compounds. The decrease in GO concentration with thermal treatment has been previously reported in the literature. Khuwijitjaru et al. [58] explored the degradation kinetics of GO when submitting RBO to temperatures from 132 °C to 222 °C, reporting that GO thermal degradation occurred at a constant rate, increasing with the heating temperature; these authors also reported differences among the individual constituents of GO regarding its thermal degradation rate. 

Toyoizumi et al. [59] reported a decrease in the GABA content and most amino acids when cooking germinated brown rice at high temperatures (105 °C, 115 °C, 125 °C, and 135 °C) for 40 min. Therefore, there was a thermal decomposition of these compounds. Le et al. [60] also reflected on the GABA content during some processing steps, including the temperature effect; the authors used a solution containing GABA and a germinated soy milk with temperatures of 70 °C and 90 °C applied for 30 min. The temperature resulted in different effects on GABA: both heated GABA solutions showed no loss of GABA content, while in the germinated soy milk, the GABA content decreased significantly with heating. These results reveal that the stability of GABA can vary with temperature but also with the food matrices. Srisang et al. [61] tested the quality of germinated brown rice after drying. They applied high drying temperatures (90 °C, 110 °C, 130 °C, and 150 °C) in two rice varieties; the results of GABA content did not significantly differ. Chungcharoen et al. [62] found the same results in germinated brown dried rice at 130 °C and 150 °C. These results reveal that temperature may positively affect the GABA content by increasing GAD enzyme activity through cell wall disruption.

The GO content in RBO also presented differences (*p* < 0.05) among varieties; however, the RBO extracted from NSRB did not show any statistically significant differences (*p* > 0.05) when compared to the RBO extracted after RB stabilisation. There was an appreciable increase in the GO content of the oils (NSRBO, SRBO) compared with their respective brans (NSRB, SRB). These results may be related to the cell wall disruption that occurred during extraction; on the other hand, they may reflect the difference between extraction techniques, as the solvent extraction with isopropanol before the RB analysis by HPLC is less efficient in terms of oil yield than the MAE extraction, as verified in the previous optimisation tests.

Table 5 presents the relative percentages of the four more abundant GO compounds for SRB, NSRB, and RBO, showing changes with stabilisation (except for Teti) and extraction conditions, mainly detected in the Luna variety. The predominance of MCAF followed by CAF is in accordance with those previously described [40]. The RB from the Luna variety shows a higher percentage of CampF (20.7 ± 1.2) than Ariete and Teti (14.5 ± 0.6 and 13.4 ± 1.3 respectively) (*p* < 0.05). Khuwijitjaru et al. [58] reported a lower degradation rate of CampF under 160 °C. CampF is more heat-stable than the other GO compounds. This fact can justify the different behaviour of the Luna variety without the corresponding decrease in total GO content after stabilisation (Table 5).

The hypothesis that GO compounds have different heat stabilities has been previously explored by Shin et al. [63], who reported that steryl ferulates were more resistant to heat than triterpenoid alcohol ferulates. These results may be the origin of the findings of Min et al. [64], who analysed the effect of parboiling, wet cooking, and a combination of both thermal treatments on the bioactive compounds of rice with different bran colours, reporting the stability or increase in the GO content depending on the cultivar. These results demonstrated that the individual components of the GO of each cultivar may affect its thermal stability by responding differently to temperature.

Although current data are limited, the oil extraction process seems to increase the ratio of SF while maintaining the CAF proportion. 

## 4. Conclusions

Here, a microwave-assisted method was assessed for RB stabilisation and optimised for oil extraction. The stabilisation process effectively decreased the acid value of bran during storage. The stabilisation decreased the GO contents in the cases of the Ariete and Teti varieties, but did not affect the Luna variety. The different GO behaviour of Luna can be justified by a higher content of the heat-stable compound campesteryl ferulate (CampF). Increased GABA levels after RB stabilisation treatment were obtained, probably due to the impact of cell wall disruption on the GAD enzymatic activity.

Maximum oil extraction yields were found at 120 °C and with ethanol as the solvent in a 9:1 solvent ratio. The stabilisation does not significantly affect the GO contents in the RBO samples.

The RBO samples showed a ratio higher than two between polyunsaturated (PUFA) and saturated fatty acids (SFA), with the Teti variety presenting the highest PUFA:SFA ratio (2.75). The amount of linolenic acid was higher than previously reported for other vegetable oils, including olive oil.

Microwaved RB stabilisation and subsequent MAE is a fast and effective way of treating and valorising relevant side streams of rice milling. The results of this study regarding the behaviour of different rice varieties under these methods, concerning the oil extraction and composition, can be useful to add value to important healthy bioactive compounds.

## Figures and Tables

**Table 1:** Water absorption capacity, moisture content, and acid values for stabilised (SRB) and non-stabilised rice bran (NSRB) at the initial (T0) and final storage times (T7-week 16), expressed as mean ± SD.

	T	Teti		Luna		Ariete	
		NSRB	SRB	NSRB	SBR	NSRB	SRB
WAC	0	132.47 ± 0.81	129.30 ± 0.70	123.03 ± 0.56	145.28 ± 5.66	130.01 ± 1.02	127.89 ± 4.13
	7	126.73 ± 2.53	182.74 ± 1.36	122.64 ± 0.60	145.87 ± 2.30	122.68 ± 1.20	148.18 ± 4.91
MC	0	11.03 ± 0.12	14.48 ± 0.32	10.80 ± 0.07	13.58 ± 0.10	10.28 ± 0.04	14.48 ± 0.32
	7	10.60 ± 0.00	15.00 ± 0.10	10.10 ± 0.10	12.50 ± 0.00	9.80 ± 0.10	16.00 ± 0.00
AV	0	695.61 ± 13.54	697.44 ± 1.39	606.5 ± 10.07	330.68 ± 0.09	614.06 ± 0.89	563.19 ± 2.33
	7	2520.8 ± 0.06	952.75 ± 6.42	1896.17 ± 21.07	513.29 ± 12.94	1823.83 ± 2.38	618.88 ± 14.26

**Table 2:** Rice bran oil (RBO) extraction yields (%) at different temperatures, solvents, and solvent-to-sample ratios. Results are expressed in mean ± SD. Different letters indicate statistically significant differences at *p* < 0.05 between temperature extractions with the same solvent and ratio; bold values indicate statistically significant differences at *p* < 0.05 between solvent ratios at the same temperature with the same solvent; significant differences at *p* < 0.05 between solvents at the same ratio and temperature are denoted with an asterisk (*).

Rice Variety	Solvent	Solvent to Sample Ratio	Temperature (°C)		
			80	100	120
Teti	Ethanol	3:1	**6.78 ± 0.41** ^b^	**6.85 ± 0.58** ^b^	**11.39 ± 0.95** *^,a^
		9:1	**8.36 ± 0.91** *^,b^	**12.91 ± 0.81** *^,b^	**18.20 ± 1.50** *^,a^
	Isopropanol	3:1	**6.42 ± 0.55** ^b^	**7.20 ± 0.60** ^b^	**10.12 ± 0.64** *^,a^
		9:1	**7.54 ± 0.38** *^,a^	**10.95 ± 0.90** *^,a^	**16.02 ± 1.10** *^,a^
Luna	Ethanol	3:1	6.71 ± 0.58 ^b^	**6.54 ± 0.51** ^b^	**10.77 ± 1.2**4 ^a^
		9:1	7.54 ± 0.94 ^a^	**12.03 ± 1.7**5 ^a^	**17.88 ± 1.78** ^a^
	Isopropanol	3:1	6.81 ± 0.67 ^a^	**7.97 ± 0.12** ^a,b^	**10.30 ± 0.37** ^b^
		9:1	6.98 ± 0.71 ^b^	**10.29 ± 0.92** ^b^	**17.27 ± 0.23** ^a^
Ariete	Ethanol	3:1	7.06 ± 0.64 ^b^	**7.31 ± 0.47** ^b^	**12.02 ± 0.82** ^a^
		9:1	7.49 ± 1.21 ^b^	**11.72 ± 0.96** *^,b^	**17.90 ± 1.19** ^a^
	Isopropanol	3:1	6.87 ± 0.71 ^b^	**7.34 ± 0.59** ^b^	**10.27 ± 0.52** ^a^
		9:1	6.94 ± 0.24 ^a^	**9.99 ± 0.95** *^,a^	**16.11 ± 0.88** ^a^

**Table 3:** Fatty acid profiles, including palmitic (C16:0) and stearic acid (C18:0), oleic (C18:1n9c), linoleic (C18:2n6c) and linolenic acids (C18:3n3c) of rice bran oil (RBO) extracted using the MAE. Saturated fatty acids (SFA), monounsaturated fatty acids (MUFA), polyunsaturated fatty acids (PUFA) as well as the ratio PUFA:SFA and the ratio omega 3 to omega 6 (ω3:ω6) are also represented. The results are expressed as % mean ± SD. Different letters indicate statistically significant differences at *p* < 0.05 in the same row.

Fatty Acid	Rice Bran Variety		
	Luna (*n* = 3)	Ariete (*n* = 3)	Teti (*n* = 3)
C16:0	16.37 ± 0.12 ^a^	15.70 ± 0.10 ^b^	13.67 ± 0.06 ^c^
C18:0	1.20 ± 0.00 ^a^	1.20 ± 0.00 ^a^	1.20 ± 0.00 ^a^
C18:1n9c	38.33 ± 0.00 ^a^	38.43 ± 0.06 ^a^	37.00 ± 0.00 ^b^
C18:2n6c	39.10 ± 0.17 ^c^	39.57 ± 0.06 ^b^	42.73 ± 0.06 ^a^
C18:3n3c	1.57 ± 0.06 ^b^	1.53 ± 0.06 ^b^	2.00 ± 0.00 ^a^
SFA	18.97 ± 0.06 ^a^	18.27± 0.12 ^b^	16.40 ± 0.00 ^c^
MUFA	39.90 ± 0.10 ^a^	39.97 ± 0.06 ^a^	38.50 ± 0.10 ^b^
PUFA	41.10 ± 0.10 ^c^	41.70 ± 0.00 ^b^	45.10 ± 0.00 ^a^
PUFA:SFA	2.17	2.28	2.75
ω3:ω6	0.041	0.038	0.046

**Table 4:** Gamma oryzanol (GO) and gamma-aminobutyric acid (GABA) contents for stabilised (SRB) and non-stabilised rice bran (NSRB) and GO contents in respective rice bran oils (RBO), expressed in mg/100g sample. The results are expressed as mean ± SD. Different letters indicate statistically significant differences at *p* < 0.05 in the same column. Bold values indicate statistically significant differences at *p* < 0.05 between SRB and NSRB.

	GO (mg/100g RB)		GABA (mg/100g RB)		GO (mg/100g RBO)	
Variety	NSRB	SRB	NSRB	SRB	NSRBO	SRBO
Ariete	**272.7 ± 16.9 ^b^**	**178.8 ± 40.9 ^b^**	**475.0 ± 45.6**	**600.7 ± 18.0 ^b^**	4684.6 ± 66.4 ^a^	4163.6 ± 467.1 ^b^
Luna	276.0 ± 7.4 ^b^	299.3 ± 2.0 ^a^	500.0 ± 34.9	549.6 ± 38.1 ^b^	4624.8 ± 217.2 ^a^	4976.2 ± 103.8 ^a^
Teti	**403.6 ± 40.2 ^a^**	**305.9 ± 63.9 ^a^**	**508.1 ± 7.3**	**747.1 ± 29.8 ^a^**	4273.4 ± 35.3 ^b^	4469.3 ± 376.5 ^a,b^

**Table 5:** Gamma oryzanol (GO) compounds: 24-methylenecycloartanyl ferulate (MCAF), cycloartenyl ferulate (CAF), campesteryl ferulate (CampF), and β-sitosteryl ferulate (SF) for stabilised (SRB) and non-stabilised rice bran (NSRB), in rice bran (RB) and rice bran oil (RBO). The results are expressed in % of total GO as mean ± SD. Values in bold indicate statistically significant differences at *p* < 0.05 between RB and RBO from the same rice variety and stabilisation procedure; significant differences at *p* < 0.05 between stabilised and non-stabilised samples from the same cultivar and sample matrix are denoted with an asterisk.

		Ariete		Luna		Teti	
		NSRB	SRB	NSRB	SRB	NSRB	SRB
CAF (%)	RB	31.5 ± 3.9	31.7 ± 6.6	26.4 * ± 1.5	36.3 * ± 1.5	30.2 ± 7.4	31.0 ± 6.3
	RBO	31.1 ± 0.6	31.3 ± 1.9	**30.2 ± 0.9**	**31.3 ± 0.8**	29.6 ± 0.7	30.0 ± 0.5
MCAF (%)	RB	**41.4 ± 2.8**	**42.1 ± 3.3**	**40.7 ± 1.2**	**38.6 ± 0.9**	**41.5 ± 1.3**	**43.1 ± 1.4**
	RBO	**33.4 ± 0.1**	**34.1 ± 1.1**	**35.5 ± 0.8**	**34.9 ± 0.9**	**36.6 ± 0.7**	**36.9 ± 0.7**
CampF (%)	RB	14.5 * ± 0.6	12.6 * ± 0.4	20.7 * ± 1.2	16.2 * ± 0.6	13.4 ± 1.3	14.9 ± 2.8
	RBO	**13.0 ± 0.1**	12.7 ± 0.5	15.9 * ± 0.1	14.9 * ± 0.1	12.0 ± 0.2	11.9 ± 0.6
SF (%)	RB	**12.7 ± 1.4**	**13.6 ± 3.8**	12.2 * ± 0.6	8.9 * ± 0.1	14.9 ± 8.8	**11.1 ± 2.3**
	RBO	**22.4 ± 0.4**	**22.0 ± 0.4**	**18.4 ± 0.3**	**18.9 ± 0.3**	21.7 ± 0.2	**21.3 ± 0.4**

## Data Availability

Data is contained within the article.
